# Color Perception and Attentional Load in Dynamic, Time-Constrained Environments

**DOI:** 10.3389/fpsyg.2018.02614

**Published:** 2019-01-08

**Authors:** Stefanie Hüttermann, Nicholas J. Smeeton, Paul R. Ford, A. Mark Williams

**Affiliations:** ^1^Cognitive and Team/Racket Sport Research, German Sport University Cologne, Cologne, Germany; ^2^Sport and Exercise Science and Medicine, University of Brighton, Brighton, United Kingdom; ^3^Department of Health, Kinesiology, and Recreation, University of Utah, Salt Lake City, UT, United States

**Keywords:** color sensitivity, cone distribution, decision making, focus of attention, football

## Abstract

The capacity to perceive color in the peripheral field has attracted research interest for more than a decade. There is evidence that sensitivity to red-green color variations is lower than for yellow-blue in peripheral vision. Whether, and to what extent, color vision affects the visual focus of attention, which is normally much smaller than the visual field, has not yet been examined. We used a sport-specific decision-making task to assess whether the color of the jersey worn by players appearing in the periphery influences decision making by affecting the attentional and perceptual capabilities. Pairs of players wearing chromatic (blue, yellow, red, and green) and achromatic (black, white) colored jerseys were briefly presented across a range of visual angles on a 6 m concave immersive screen. Participants were required to decide to whom to pass and recall relevant information. Findings indicate that color perception changes vary with increasing visual angle, but that the focus of attention is too small to be influenced by jersey color sensitivity. Decision-making performance decreases with increasing visual angle, but is not influenced by color. The implications for decision-making processes in sport and other professional domains are discussed.

## Introduction

It is well reported that color perception changes across the visual field (Newton and Eskew, 2003; McKeefry et al., 2007; Hansen et al., 2009). Usually, it is best in the fovea (central vision) and declines in the periphery. While most researchers have concentrated on visual perception, the effect of different colored stimuli on people’s focus of attention, which normally is much smaller than the visual field (for a review, see Hüttermann and Memmert, 2017), has not been adequately investigated. In the current study, this issue was dealt with by using sport-specific game situations in which visual attention and the ability to distinguish different colors play an important role.

Several researchers have examined whether jersey color influences performance in sportspeople (e.g., Hill and Barton, 2005; Greenlees et al., 2008; Adam and Galinsky, 2012). Overall, performance in these studies provide evidence that color influences people’s attitudes and behavior. For example, people are more likely to behave aggressively when wearing black compared to white jerseys (cf., Frank and Gilovich, 1988). Moreover, athletes in combat sports (e.g., boxing, tae kwon do, wrestling) at the Olympic Games in 2004 who were wearing red jerseys were more often successful than those wearing blue jerseys (Hill and Barton, 2005). Findings support previous research showing that colors elicit certain unique psychological properties and can have a strong impact on emotional feelings (Hemphill, 1996). Thus, red stimuli, for example, are usually perceived as dominant and cause negative effects in those viewing them (Attrill et al., 2008). However, researchers have mainly focused on the effect of jersey color on judging aggressiveness, chances of winning, and fairness. Only one study by Olde Rikkert et al. (2015) has focused on the effects of outfit color on the assessment of players’ positions. Their analysis revealed the widest angle of perception and location assessment was for players wearing white when compared to other colored jerseys. These findings can be explained by differentiation of the characteristics of chromatic and achromatic colors in visual periphery.

In general, colors can broadly be divided into chromatic and achromatic colors (Valdez and Mehrabian, 1994). Black, white, and the various shades of gray are achromatic colors. These colors have lightness, but no hues (all wavelengths are present in equal amounts within those colors). In contrast, any color in which one particular wavelength or hue dominates is called a chromatic color. Blue, yellow, red, and green are chromatic colors. Human color vision is characterized by photoreceptor cells in the retina consisting of two cone-opponent mechanisms, referred to as red-green (distinguish between L- and M-cone responses) and blue-yellow (differences with a combination of L- and M-cones; Mullen et al., 2005). Sensitivity to red-green color variations declines less toward the periphery than sensitivity to luminance or blue-yellow colors. This decline can be explained by the increasing size of receptive fields of parvocellular retinal ganglion cells, as well as the unselective or random contribution of L- and M-cones to the surrounding receptive field (Martin et al., 2001). Researchers have revealed inconsistencies in the eccentricities (i.e., distance to fovea in degrees of visual angle) up to which L and M cone opponency becomes absent. Mullen et al. (2005), for example, found that L/M cone opponency has no impact on behavior at eccentricities of 25–30 degrees (in the nasal field). Martin et al. (2001) suggest that color vision/detection declines with increasing eccentricity, but it is still possible even at large eccentricities (up to 50 degrees). While it is clear how these physiological differences across the retina might affect people’s visual field for color perception, the correspondence with people’s ability to attend to objects with different colors in the periphery is less clear.

Visual focus of attention is typically allocated across part of the visual field. Previously, researchers have shown that visual attention is a prerequisite for conscious recognition of information. In general, people only consciously perceive those objects/events onto which they direct their attention at a given time (Dehaene et al., 2006). During the past few decades, various methods/paradigms have been developed to measure spatial attention (e.g., cueing, flanker interference, crowding, counting tasks). Due to the diversity of these tasks, outcomes are inconsistent and difficult to compare (for a review, see Intriligator and Cavanagh, 2001). Hüttermann et al. (2013) developed an attention-demanding task (attention-window task) determining the maximum size of the attentional focus when two objects are presented in the visual periphery. All studies using this task have confirmed that the attentional focus is smaller than the visual field (for a review, see Hüttermann and Memmert, 2017). Due to attentional width be no greater than visual angles of 30–45 degrees (depending on age and expertise group; cf. Hüttermann et al., 2014) and scientific analyses showing that color vision declines with eccentricity (distance from fixation) above 50 degrees (Martin et al., 2001), it can be assumed that the physiological limitations of color detection on the retina do not influence color detection in the range of visual angles found during focus of attention tasks.

There are many real-life situations, such as when driving or playing sport, in which good visual attentional skills play an important role during decision making. In complex team sports, for example, players who possess superior attentional capability are able to include a higher frequency of relevant players in their decision-making process (Williams et al., 1999). While many researchers have assessed decision making and perceptual and attentional capabilities in athletes, the potential impact of color (i.e., jersey color) has not yet been investigated. Olde Rikkert et al. (2015) found an effect of color selection on peripheral vision, yet there is no published study examining the impact of color on visual attention related to decision making. However, in team sports, a wide attentional focus (attention window) is required in conjunction with high levels of perceptual-cognitive skill (cf., Hüttermann et al., 2014), especially when sports are played on pitches and courts where players are dispersed across a large visual angle (e.g., football).

In the current study, we used a football-specific decision-making task to assess whether jersey color affects decision making as a function of attentional and perceptual capabilities. As per the attention-window task used by Hüttermann et al. (2013), participants were required to judge two stimuli equidistant to the center of their visual field on their left and right side with varying separations between stimuli. Stimuli were teammates and opponent players wearing either black and white jerseys (achromatic colors), red and green jerseys (chromatic colors), or blue and yellow jerseys (chromatic colors). An attentional task required the differentiation between color and shape of stimuli (recognition of players wearing black jerseys and assessment of their running direction) so that it demanded visual attention (cf., Treisman and Gelade, 1980). A perceptual task required only the differentiation between jersey colors (recognition of number of players wearing white jerseys) so that it was a recognition rather than an attention-demanding task. A decision-making task required the selection of a pass with the ball to “open” teammates or not. We expected wider angles between stimuli to be negatively related to performance. We assumed that color does not negatively affect the size of people’s focus of attention as it is usually smaller than 50 degrees of visual angle (e.g., Martin et al., 2001; Hüttermann et al., 2014). We expected to observe, based on research showing differences in acuity in the visual periphery between chromatic and achromatic colors (e.g., Mullen et al., 2005), differences between these two color groups. Moreover, because researchers have reported sensitivity to red-green variations is lower than to blue-yellow colors in the periphery (e.g., Nagy and Wolf, 1993), we expected higher perceptual capabilities when players in the periphery (visual angles greater than 50°) wore yellow-blue than red-green jerseys. In contrast to many other studies investigating perceptual and attentional capabilities oftentimes using small screens, our study was performed using a large immersive dome screen (creating immersive 210° stimulus projection environment). This relatively novel approach allowed us to more realistically measure perceptual and attentional skills related to decision making across a broader field of view.

## Materials and Methods

### Participants

Altogether, 20 participants (4 female) aged 21–26 years (*M*_age_ = 23.55 years, *SD* = 1.73 years) took part. Data from one participant were excluded due to low math accuracy (<85%) on the Aospan task (cf., Unsworth et al., 2005). At the time of data collection, participants regularly took part in a team sport. Primary sports included basketball (*n* = 3), cricket (*n* = 2), football (*n* = 9), lacrosse (*n* = 3), and netball (*n* = 3). Participants reported normal or corrected-to-normal (with contact lenses) vision. Wearers of glasses had to be excluded as their whole visual field is usually not covered by glasses. The study was carried out in accordance with the Helsinki Declaration of 1975 and written informed consent was obtained from each participant prior to testing. Approval was obtained from the lead institution’s ethics board.

#### Football-Specific Decision-Making Task

**FIGURE 1:**
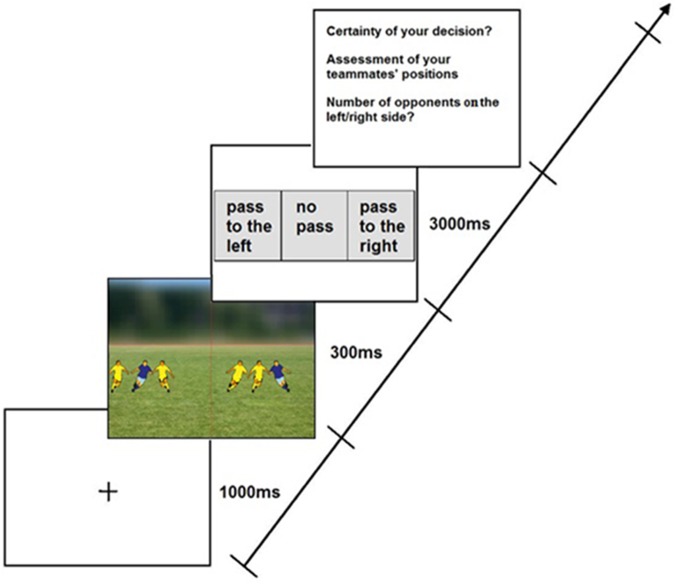
Sequence of events in one exemplary trial showing a game situation with teammates wearing blue jerseys and opponent players yellow jerseys.

**FIGURE 2:**
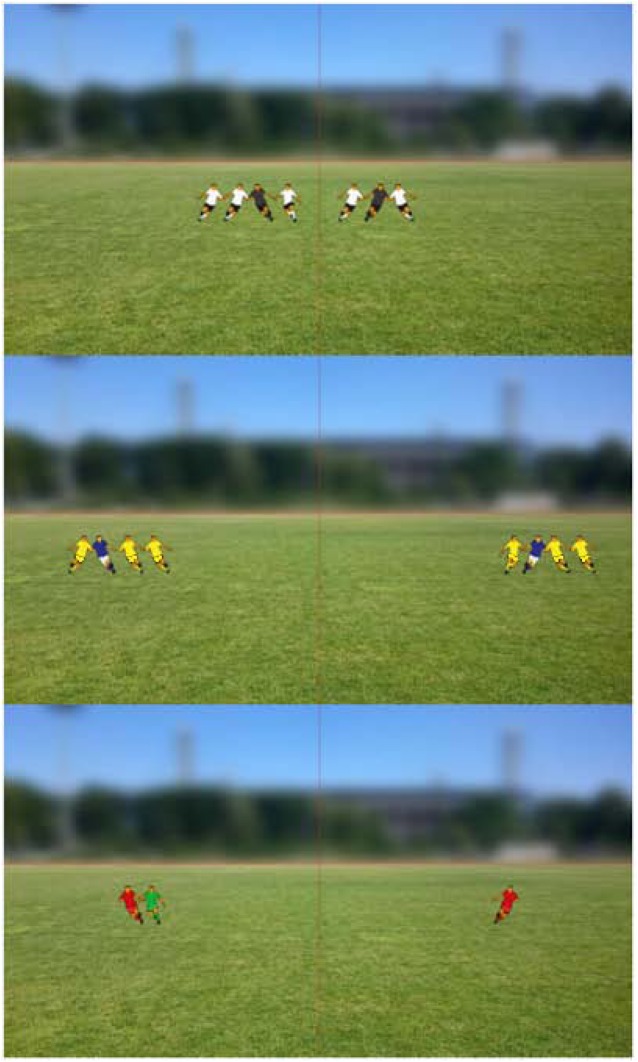
A representation of three exemplary trials showing the teammates in black (upper picture; blue: middle picture; red: bottom picture) jerseys and the opponent players in white (upper picture; yellow: middle picture; green: bottom picture) jerseys. Participants should decide to pass the ball in none of these situations as the teammates are either running toward the side lines or are surrounded by opponent players.

This task was presented using Delphi XE 3. Participants completed three versions/conditions of this task in a randomized order that differed only in the color of stimuli (i.e., color of teammate and opponent jerseys). In each of the three conditions, participants performed 24 trials preceded by 2 additional practice trials. At the beginning of each trial, a central fixation cross (1000 ms) appeared, followed by the presentation of two stimuli for 300 ms equidistant and on opposite sides from the fixation cross (see Figure 1). Stimuli were randomly presented at one of eight horizontal distances from the center of the immersive screen (20°, 40°, 60°, 80°, 100°, 120°, 140°, and 160°; note that these visual angles represent the total observation angle (i.e., the summed eccentricity on each side of the participant’s field of vision) and were equally likely to appear at each visual angle. The stimuli consisted of different player configurations (the players’ height was approximately 30 cm) including one teammate surrounded by zero, one, two, or three opposing players (randomly either on his right or left side). While the opposing players always moved toward the respective teammate on each side of the participant, the teammate could either move in the direction toward the center of the screen or toward the sideline (outer end of the screen). Figure 2 shows three exemplary trials with the opponent players and teammates wearing different colored jerseys. As participants have to detect the conjunction of both form (direction of the teammates’ movement: toward the center versus toward the sideline) and coloring (different colored jerseys of teammates and opponents) of the stimuli, the task is classified as attention-demanding (cf., Treisman and Gelade, 1980).

When standing in front of the immersive dome (IGLOO Vision Ltd., Shropshire, United Kingdom), participants were required to imagine they were the player in possession of the ball and to decide whether it would be best to pass the ball to one teammate or to stop/control the ball (decision-making task). They were requested to decide to pass the ball only to the left or right side if they perceived a teammate was running in their direction (toward the center) and was not surrounded by an opponent player. If a teammate was running toward the side line and/or was surrounded by at least one opponent player, participants should decide not to pass the ball. Participants were asked to verbally report their decision (pass to the left, pass to the right, no pass) quickly and accuracy, but at least within a time limit of 3 s. Afterward, they were required to report how certain they were about their decision on a ten-point Likert scale ranging from 1 (very uncertain) to 10 (very certain). Subsequently, they specified the teammates’ running direction for each side (attention task) and the number of opponent players surrounding their teammate (perceptual task), as well as reporting their certainty level using the Likert scale.

#### Automated Operation Span (Aospan) Task

The Aospan task was programmed and run in E-Prime 2.0 (Psychology Software Tools, Pittsburgh, PA, United States). In this task, participants memorized lists of letters (e.g., NYK; PQLRSFT) while at the same time solving simple mathematical problems (e.g., 3 × 3 = ?; 20-4 = ?) (Unsworth et al., 2005). In total, the Aospan task included 15 trials (3 trials each with 3, 4, 5, 6, and 7 letters to memorize). Participants were informed about the need to maintain their math accuracy at or above 85% at all times, as the operation span score was only valid if participants were above this threshold at the end of the task. The dual-task (math/memory) should place a burden on limited-capacity executive attention resources (Conway et al., 2005). In line with the standard procedure concerning the data evaluation (cf., Unsworth et al., 2005), we used the total number of letters recalled across all error-free trials as a measure of working memory.

### Procedure

**FIGURE 3:**
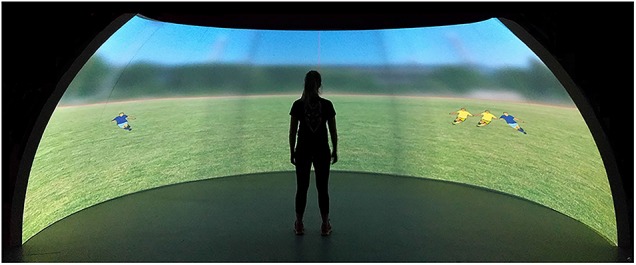
The figure shows the experimental setup with a participant standing in front of the 2.4 m × 6 m IGLOO dome and completing the test condition with players wearing blue (teammates) and yellow (opponents) jerseys.

In randomized order, participants performed one of the three versions of the football-specific task (black-white jerseys, red-green jerseys, blue-yellow jerseys) and the Aospan task once (cf., Unsworth et al., 2005). They were tested individually in a laboratory room. For the implementation of the football-specific tasks, participants stood approximately 3 m from a 210° curved projection screen (IGLOO, radius of 3 m, height: 2.20 m; see Figure 3). The implementation of the Aospan task was carried out sitting with a distance of approximately 50cm in front of a 50 13-inch display (resolution: 1366 × 768 pixels). Instructions were delivered on the screen and participants were encouraged to ask questions prior to starting.

## Results

### Total Score

**FIGURE 4:**
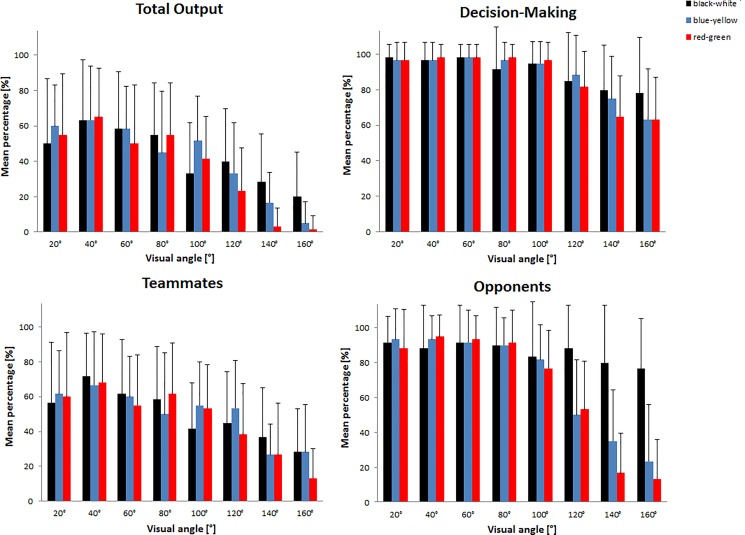
Percentage of participants’ total accuracy rate, their decision making, the identification rate of the teammates’ running direction, and the identification rate of the number of opponents in the football decision-making task, in degrees of visual angle as a function of jersey color (black-white, blue-yellow, and red-green). Symbols represent across-participant means, and error bars show standard deviations.

In the football decision-making tasks, responses were only counted as correct if participants made the right decision whether to, and where, to pass the ball, correctly identifying the running direction of both teammates, and reporting the right number of opponent players on both sides of the screen. In total, participants correctly evaluated 40.69% (*SD* = 6.45%) trials. We conducted a repeated-measures ANOVA with accuracy rate as the dependent variable and visual angle (20°, 40°, 60°, 80°, 100°, 120°, 140°, and 160°) plus jersey color (black-white, red-green, blue-yellow) as the within-participant factors. The descriptive data are presented in Figure 4.

The ANOVA revealed that accuracy rate differed as a function of visual angle, *F*(7,133) = 30.994, *p* < 0.001, η^2^ = 0.620, with accuracy decreasing with increasing visual angles. There was no effect of color, *F*(2,38) = 1.698, *p* = 0.197, but there was a Color × Angle interaction, *F*(14,266) = 1.733, *p* = 0.049, η^2^ = 0.084. We collapsed the data into smaller (20°, 40°, 60°, and 80°) and larger angles (100°, 120°, 140°, and 160°) and performed a contrast analysis comparing performances between achromatic (black-white) and chromatic (red-green, blue-yellow) colored jerseys. Participants reached higher accuracy rates at smaller angles (20°, 40°, 60°, and 80°) when players wore chromatic colored jerseys compared to achromatic colored jerseys, *t*(19) = 4.699, *p* < 0.001, *d* = 1.05. In contrast, accuracy rates were higher for larger angles (100°, 120°, 140°, and 160°) when players wore achromatic colors compared to chromatic colors, *t*(19) = 10.589, *p* < 0.001, *d* = 2.37.

### Decision Making

We conducted a repeated-measures ANOVA with accuracy rate for decision making (pass to the left, to the right, or no pass) as the dependent variable and visual angle and jersey color as the within-participant factors. Since Mauchly’s test revealed violations of the sphericity assumption for color, χ^2^(2) = 8.762, *p* = 0.013, angle, χ^2^(27) = 65.081, *p* < 0.001, and Color × Angle factors, χ^2^(104) = 190.308, *p* < 0.001, we used adjusted degrees of freedom based on the Greenhouse-Geisser correction. For analyses in which the sphericity assumption was violated, we reported the value of ε from the Greenhouse-Geisser correction. The ANOVA revealed that angle influenced decision making, *F*(3.149,59.831) = 30.618, *p* < 0.001, η^2^ = 0.617, ε = 0.450, with higher accuracy rates at smaller (20°, 40°, 60°, and 80°) when compared to greater angles (100°, 120°, 140°, and 160°). There was neither a main effect of color, *F*(1.444,27.429) = 0.684, *p* = 0.467, ε = 0.722, nor a significant interaction between Color × Angle, *F*(5.535,105.160) = 1.461, *p* = 0.203, ε = 0.395. Furthermore, we analyzed certainty rates (i.e., how sure participants were about their decisions) using a Likert scale. In total, participants reported a certainty value of 7.44 (*SD* = 0.36). A repeated measures ANOVA with jersey color as the within-participant factor revealed no significant differences between the confidence ratings across the different jersey colors, *F*(1.254,23.834) = 2.682, *p* = 0.108, ε = 0.627 (Mauchly’s test of sphericity: χ^2^(2) = 16.242, *p* < 0.001).

### Attention

We performed a further ANOVA with the same within-participant factors to analyze the accuracy of identification of the teammates running direction (attentional task). The ANOVA revealed a significant main effect of angle, *F*(7,133) = 17.902, *p* < 0.001, η^2^ = 0.485, indicating that participants were better able to solve the attentional task with smaller angles (20°, 40°, 60°, and 80°) between stimuli than with greater angles (100°, 120°, 140°, 160°). There was neither a main effect of color, *F*(2,38) = 0.556, *p* = 0.578, nor a Color × Angle interaction, *F*(14,266) = 0.967, *p* = 0.488. In addition, we analyzed participant certainty rates relating to their perception of the running direction of teammates. On average, they reported a confidence value of 5.44 (*SD* = 0.61). A repeated measures ANOVA with jersey color as the within-participant factor did not reveal any differences between the confidence ratings across the different jersey colors, *F*(2,38) = 2.046, *p* = 0.143.

### Perception

To analyze the accuracy of identification for the number of opponent players (perceptual task), we conducted a further ANOVA with the same factors as before. Since Mauchly’s test revealed violations of the sphericity assumption for both color, χ^2^(2) = 18.952, *p* < 0.001, and Color × Angle, χ^2^(104) = 147.295, *p* = 0.009, we used adjusted degrees of freedom based on the Greenhouse-Geisser correction. Participants identified the number of players more often correctly for smaller (20°, 40°, 60°, and 80°) compared with greater angles (100°, 120°, 140°, and 160°?), *F*(7,133) = 61.689, *p* < 0.001, *η*^2^ = 0.765. There was a significant main effect of color, *F*(1.211,23.015) = 14.743, *p* < 0.001, η^2^ = 0.437, ε = 0.606, as well as a significant interaction effect between color and angle, *F*(6.267,119.068) = 11.302, *p* < 0.001, η^2^ = 0.373, ε = 0.448. Participants reported the number of opponent players more often wearing white jerseys compared to yellow, *t*(19) = 3.275, *p* = 0.004, *d* = 0.73, and to green, *t*(19) = 4.760, *p* < 0.001, *d* = 1.06, with no difference between yellow and green jerseys, *t*(19) = 1.940, *p* = 0.067. Summarizing visual angles in two categories (small angles: 20°, 40°, 60°, and 80°; great angles: 100°, 120°, 140°, and 160°), we found no difference in the accuracy rate between small and large angles for the color white, *t*(19) = 1.795, *p* = 0.089, but lower accuracy scores were reported at larger angles for the colors yellow, *t*(19) = 8.389, *p* < 0.001, *d* = 1.88, and green, *t*(19) = 12.730, *p* < 0.001, *d* = 2.85, indicating that color perception changes across the visual field. Furthermore, we analyzed participant certainty rates. In total, they reported a confidence value of 6.25 (*SD* = 0.49). A repeated-measures ANOVA with color as the within-participant factor revealed a difference between the confidence ratings dependent on jersey color, *F*(1.506,28.606) = 19.203, *p* < 0.001, η^2^ = 0.503, ε = 0.753 (Mauchly’s test of sphericity: χ^2^(2) = 7.165, *p* = 0.028. Participants were more confident about their responses when they had to count the number of opponent players wearing white jerseys compared to yellow, *t*(19) = 3.640, *p* = 0.002, *d* = 0.835, and green jerseys, *t*(19) = 5.318, *p* < 0.001, *d* = 1.220. Furthermore, they were more confident in their counting report of opponent players wearing yellow compared to green jerseys, *t*(19) = 3.023, *p* = 0.007, *d* = 0.694.

### Aospan Task

In the Aospan task, participants achieved an average score of 64.25 (*SD* = 5.68) out of a possible total value of 75. There was no significant correlation between accuracy on the football decision-making task and performance (average score) on the Aospan task (*r* = 0.260, *p* = 0.268).

## Discussion

The capacity to perceive color in the visual periphery has been a subject of investigation for several decades. It is best in central vision and far less sensitive in the periphery. In the current study, we examined for the first time whether different-colored jerseys in team sports affect field of perception, attentional focus (attention window), and decision making in football-specific game situations. Our findings indicate that coloring does affect the size of the visual field, but does not affect attentional focus or decision making in game situations. As we did not find a positive correlation between performance on the football task and a working memory task (Aospan task; cf. Unsworth et al., 2005), the results from the football task can therefore be attributed to attentional and perceptual capabilities, rather than working memory capacity. Findings confirm previous research demonstrating that achromatic, but not chromatic colors of jerseys facilitate the perception of player positioning in the periphery (cf., Olde Rikkert et al., 2015). Additionally, it expands on existing research by showing that jersey color does not affect attentional capabilities or decision making.

While previously researchers have shown that the visual field is much greater than attentional focus (for a review, see Hüttermann and Memmert, 2017), the attentional focus seems to be too small to be influenced by color change perception. In the current study, the players were able to extend their attention over visual angles of about 100° without a significant decline in performance. Moreover, we observed awareness limitations independently of color. This latter finding supports previous research suggesting that color vision declines with increasing eccentricity; yet color vision is still possible at eccentricities up to 50 degrees (i.e., visual angles up to 100 degrees; Martin et al., 2001). In the perception task, participants were able to correctly identify stimuli up to 100 degrees of visual angle without significant performance decrements in the blue-yellow and red-green conditions, but they were able to perform the task at wider angles without any significant losses of performance in the black-white condition. This finding supports previous research showing that color perception changes across the visual field (e.g., Hansen et al., 2009) and that there are differences between chromatic and achromatic colors (e.g., Nagy and Wolf, 1993).

Overall, our assumptions confirm that stimulus color affects athletes’ perceptual capabilities in the visual periphery, while at the same time it does not having a negative influence on the size of their attentional focus. This finding can be explained by the fact that the focus of attention (i.e., the area of the human visual field in which objects/processes can be consciously perceived) is much smaller than the visual field. Additionally, we found the color of players’ jerseys did not affect decision making, even though information from players wearing colored jerseys cannot be perceived as well as players wearing colorless jerseys in the visual periphery. This important insight can provide information about the inconsistent findings in color research in sport identified by Dijkstra et al. (2018). Our results confirm and extend the findings of Dijkstra et al. (2018) by showing that there is no color effect when stimuli (in our case football players) are closer together, which is explained by the size of the attentional focus, which does not depend on stimulus color. Our data support those of researchers who have shown a close relationship between attentional capabilities and decision making in sport (e.g., Hüttermann et al., 2017, 2018). It appears that while it is not possible to perceive all information in the peripheral field in detail (e.g., the positioning of players; Olde Rikkert et al., 2015), decision making is not negatively affected as color does not have an impact on the attentional focus. We conclude that there is no need for players and coaches to think about selecting a particular jersey color to improve decision making. However, if players want to perceive more players in the visual periphery, we agree with Olde Rikkert et al. (2015) that achromatic jersey colors, such as white, are recommended.

We prioritized the replication of a number of football-specific aspects of the task, for example, we used a representative viewing perspective that was typically used by a player during match-play and a large concave immersive screen to increase the sense of presence in the environment. However, it is important to acknowledge that our findings could be different if we traded off task realism for greater control over the color perception parameters. For example, a different background may have provided a different color contrast with the jersey colors and changed the sensitivity of the perceptual system. Furthermore, we did not measure brightness/lightness effects. Provisional work in this area has found garment patterning and luminosity to influence decision making (e.g., Causer et al., 2013; Causer and Williams, 2015; Smeeton et al., 2018). A potential avenue for future research could be directed toward the HSL (hue, saturation, lightness) model (Smith, 1978). The model deals with the color type, such as red, blue, or yellow, the variation of the color depending in the lightness, and their luminance or intensity. Furthermore, in future researchers could ask participants to wear the appropriate jersey color in order to better identify with the teammates presented on video. Another potential avenue of investigation could involve further replication of the task demands, such as the integration of dynamic game scenes instead of static pictures and the effect of various stressors such as anxiety and physical workload.

In sum, we examined the extent to which color vision affects perception, attention, and decision making using a sport-specific task. Pairs of players wearing chromatic and achromatic colored jerseys were briefly presented across a range of visual angles on a large immersive screen and participants’ perception, attention, and decision making were recorded. It was concluded that the accuracy of perception of players’ jersey color differs between achromatic and chromatic colors and this effect is dependent on the visual angle at which the stimulus is presented. Overall, it appears that the color of the jerseys worn by players did not directly influence decision making or the allocation of visual attention in our simulation of football-specific scenarios.

## Author Contributions

SH, NS, and PF developed the study concept and contributed to the design. SH collected the data and analyzed it in collaboration with NS. SH wrote the first draft of the manuscript. NS, PF, and AW helped to edit and revise the manuscript. All authors approved the final submitted version of the manuscript.

## Conflict of Interest Statement

The authors declare that the research was conducted in the absence of any commercial or financial relationships that could be construed as a potential conflict of interest.
